# Supplementary material for: Quantitative determinants of aerobic glycolysis identify flux through the enzyme GAPDH as a limiting step
Source: eLife. 2014 Jul 9;3:e03342. doi: 10.7554/eLife.03342 (PMC4118620; doi:10.7554/eLife.03342)
Supplement: Supplementary file 1. — Equations and parameters used for the glycolysis model. DOI: http://dx.doi.org/10.7554/eLife.03342.009 [file elife03342s001.docx]

**Supplementary material and methods**

**Kinetic mass-balance equations**

Intracellular metabolites:

$\frac{d{GLC}_{i}}{dt}= J_{gtr}-V_{hk}$

$\frac{dG6P}{dt}= V_{hk} -V_{gpi}-V_{gsyn} -V_{g6pd}+V_{gphos}$

$\frac{dF6P}{dt}= V_{gpi} -V_{pfk}$

$\frac{dFBP}{dt}= V_{pfk} -V_{ald}$

$\frac{dGA3P}{dt}= V_{ald} -V_{gapdh}+V_{tpi}$

$\frac{dDHAP}{dt}= V_{ald} -V_{tpi}-V_{grpdh} -V_{gps}$

$\frac{d13BPG}{dt}= V_{gapdh} -V_{pgk}$

$\frac{d3PG}{dt}= V_{pgk} -V_{pgm}-V_{pgdh}$

$\frac{d2PG}{dt}= V_{pgm} -V_{en}$

$\frac{dPEP}{dt}= V_{en} -V_{pk}$

$\frac{dPYR}{dt}= V_{pk} -V_{oxphos}-V_{ldh}-V_{alt}$

$\frac{dLACi}{dt}= V_{ldh} -J_{ltr}$

$\frac{dATP}{dt}= {-V}_{hk} {-V}_{pfk}{+V}_{pgk}+V_{pk}+n_{op}V_{oxphos}-V_{atpase}+V_{ck}-V_{ak}+n_{fad}V_{gps}+n_{nad}V_{mas}$

$\frac{dADP}{dt}= V_{hk} {+V}_{pfk}{-V}_{pgk}-V_{pk}-n_{op}V_{oxphos}+V_{atpase}-V_{ck}+2\cdot V_{ak}-n_{fad}V_{gps}-n_{nad}V_{mas}$

$\frac{dP_{i}}{dt}= {-V}_{gapdh}+V_{pgdh}-n_{op}V_{oxphos}+V_{atpase}-n_{nad}V_{mas}+2\cdot V_{gsyn} {-V}_{gphos}$

$\frac{dNADH}{dt}= V_{gapdh}-V_{ldh}+V_{pgdh}-V_{grpdh}-V_{mas} {-V}_{gps}$

$\frac{dPCr}{dt}= -V_{ck}$

$\frac{dO_{2i}}{dt}= J_{O2}-{n_{ox}V}_{oxphos}$

$\frac{dGLG}{dt}= V_{gsyn} -V_{gphos}$

$\frac{dSER}{dt}= V_{pgdh} -V_{ghmt}-V_{spt}$

$\frac{dGLY}{dt}= V_{ghmt} -V_{gpt}$ ;

Extracellular metabolites:

$\frac{d{GLC}_{e}}{dt}= 0$

$\frac{dLACe}{dt}= r_{ie}\cdot J_{ltr}$

Rate laws for each flux

1. Glucose transport

$J_{gtr}=\frac{V{max}^{tr}(\frac{{Glc}_{e}}{Kmglc}-\frac{{Glc}_{i}}{Kmglc})}{1+\frac{{Glc}_{e}}{Kmglc}+\frac{{Glc}_{i}}{Kmglc}}$

1. Glucose utilization

$V_{hk}=\left( \frac{V_{fhk}^{max}\frac{{GLC}_{i}*ATP}{Kmhkf}-V_{rhk}^{max}\frac{G6P*ADP}{Kmhkr}}{1+\frac{{GLC}_{i}*ATP}{Kmhkf}+\frac{G6P*ADP}{Kmhkr}} \right)*\frac{K_{hk}^{i}}{\left( K_{hk}^{i}+G6P \right)}$

1. Phosphoglucose isomerase activity
   1. $V_{pgi}=\left( \frac{V_{fpgi}^{max}\frac{G6P}{Kmpgif}-V_{rpgi}^{max}\frac{F6P}{Kmpgir}}{1+\frac{G6P}{Kmpgif}+\frac{F6P}{Kmpgir}} \right)$
2. Phosphofructokinase

$V_{pfk}=\left( \frac{V_{fpfk}^{max}\frac{F6P*ATP}{Kmpfkf}-V_{rpfk}^{max}\frac{F16BP*ADP{*H}^{+}}{Kmpfkr}}{1+\frac{F6P*ATP}{Kmpfkf}+\frac{F16BP*ADP{*H}^{+}}{Kmpfkr}} \right)*\frac{AMP}{\left( K_{pfk}^{a}+AMP \right)}$

1. Aldolase

$V_{al}=\frac{V_{fal}^{max}\frac{F16BP}{Kmalf}-V_{ral}^{max}\frac{GAP*DHAP}{Kmalr}}{1+\frac{F16BP}{Kmalf}+\frac{GAP*DHAP}{Kmalr}}$

1. Triosephosphate isomerase

$V_{tpi}=\frac{V_{ftpi}^{max}\frac{DHAP}{Kmtpif}-V_{rtpi}^{max}\frac{GAP}{Kmtpir}}{1+\frac{DHAP}{Kmtpif}+\frac{GAP}{Kmtpir}}$

1. Glyceraldehyde phosphate dehydrogenase

$V_{gapdh}=\frac{V_{fgapdh}^{max}\frac{GAP*P_{i}*{NAD}^{+}}{Kmgapdhf}-V_{rgapdh}^{max}\frac{13BPG*NADH{*H}^{+}}{Kmgapdhr}}{1+\frac{GAP}{K_{mgap}^{gapdh}}+\frac{P_{i}}{Kmpi}+\frac{{NAD}^{+}}{K_{mnad}^{gapdh}}+\frac{GAP*P_{i}*{NAD}^{+}}{Kmgapdhf}+\frac{13BPG}{Kmbpg}+\frac{NADH}{K_{mnadh}^{gapdh}}+\frac{13BPG*NADH{*H}^{+}}{Kmgapdhr}}$ ,

1. Phosphoglycerate kinase

$$V_{pgk}=\frac{V_{fpgk}^{max}\frac{13BPG*ADP}{Kmpgkf}-V_{rpgk}^{max}\frac{3PG*ATP}{Kmpgkr}}{1+\frac{13BPG*ADP}{Kmpgkf}+\frac{3PG*ATP}{Kmpgkr}}$$

1. Phosphoglycerate mutase

$V_{pgm}=\frac{V_{fpgm}^{max}\frac{3PG}{Kmpgmf}-V_{rpgm}^{max}\frac{2PG}{Kmpgmr}}{1+\frac{3PG}{Kmpgmf}+\frac{2PG}{Kmpgmr}}$

1. Enolase

$V_{en}=\frac{V_{fen}^{max}\frac{2PG}{Kmenf}-V_{ren}^{max}\frac{PEP}{Kmenr}}{1+\frac{2PG}{Kmenf}+\frac{PEP}{Kmenr}}$

1. Pyruvate kinase

$$V_{pk}=\left( \frac{V_{fpk}^{max}\frac{PEP*ADP}{Kmpkf}-V_{rpk}^{max}\frac{PYR*ATP}{Kmpkr}}{1+\frac{PEP*ADP}{Kmpkf}+\frac{PYR*ATP}{Kmpkr}} \right)*\frac{F16BP}{\left( K_{pk}^{a}+F16BP \right)}$$

1. Lactate dehydrogenase

$V_{ldh}=\frac{V_{fldh}^{max}\frac{PYR*NADH}{Kmldhf}-V_{rldh}^{max}\frac{L{AC}_{i}*{NAD}^{+}}{Kmldhr}}{1+\frac{PYR}{K_{mpyr}^{ldh}}+\frac{NADH}{K_{mnadh}^{ldh}}+\frac{PYR*NADH}{Kmldhf}+\frac{L{AC}_{i}}{K_{mlac}^{ldh}}+\frac{{NAD}^{+}}{K_{mnad}^{ldh}}+\frac{L{AC}_{i}*{NAD}^{+}}{Kmldhr}}$ ;

1. Lactate Transport

$J_{ltr}=\frac{V_{ltr}^{max}(\frac{{LAC}_{e}}{Kmlac}-\frac{{LAC}_{i}}{Kmlac})}{1+\frac{{LAC}_{e}}{Kmlac}+\frac{{LAC}_{i}}{Kmlac}}$

1. OxPhos

$V_{ox}=\frac{V_{ox}^{max}\frac{PYR}{Kmpyrox}}{1+\frac{PYR}{Kmpyrox}}$ *$\frac{\frac{O_{2i}}{Kmo2i}}{1+\frac{O_{2i}}{Kmo2i}}*\frac{\frac{ADP}{Kmadpox}}{1+\frac{ADP}{Kmadpox}}$

1. Malate-Aspartate shuttle

$Vmas=V_{ox}=\frac{V_{ox}^{max}\frac{PYR}{Kmpyrox}}{1+\frac{PYR}{Kmpyrox}}*\frac{\frac{O_{2i}}{Kmo2i}}{1+\frac{O_{2i}}{Kmo2i}}*\frac{\frac{ADP}{Kmadpox}}{1+\frac{ADP}{Kmadpox}}$

1. ATPase

$V_{atpase}=\frac{V_{fatp}^{max}\frac{ATP}{Kmatpf}-V_{ratp}^{max}\frac{ADP*P_{i}*H^{+}}{Kmatpr}}{1+\frac{ATP}{Kmatpf}+\frac{ADP*P_{i}*H^{+}}{Kmatpr}}$

1. Creatine kinase

$V_{ck}=\frac{V_{fck}^{max}\frac{PCR*ADP}{Kmckf}-V_{rck}^{max}\frac{CR*ATP}{Kmckr}}{1+\frac{PCR*ADP}{Kmckf}+\frac{CR*ATP}{Kmckr}}$

1. Adenylate kinase

$V_{ak}=\frac{V_{fak}^{max}\frac{ATP*AMP}{Kmakf}-V_{rak}^{max}\frac{{ADP}^{2}}{Kmakr}}{1+\frac{ATP*AMP}{Kmakf}+\frac{{ADP}^{2}}{Kmakr}}$

1. Oxygen transport to the cell

$J_{O_{2}}^{tr}=k_{O_{2}}\left( C_{O2e}-C_{O2i} \right)$

1. De novo synthesis of Serine through 3-phosphoglycerate dehydrogenase (PHGDH). This involves a lumping of 3 reactions 3PG + NAD^+^ ↔ p-hPYR + NADH+H^+^, p-hPYR↔pSER, pSER↔SER

$$V_{phgdh}=\frac{V_{fphgdh}^{max}\frac{3PG*{NAD}^{+}}{Kmphgdhf}-V_{rphgdh}^{max}\frac{SER*NADH*H^{+}}{Kmphgdhr}}{1+\frac{3PG}{K_{m3pg}^{phgdh}}+\frac{{NAD}^{+}}{K_{mnad}^{phgdh}}+\frac{3PG*{NAD}^{+}}{Kmphgdhf}+\frac{SER}{K_{mser}^{phgdh}}+\frac{NADH}{K_{mnadh}^{phgdh}}+\frac{SER*NADH*H^{+}}{Kmphgdhr}}$$

1. De novo GLY synthesis through glycine hydroxymethyltransferase (GHMT, 2.1.2.1). SER↔GLY

$V_{ghmt}=\frac{V_{fghmt}^{max}\frac{SER}{Kmghmtf}-V_{rghmt}^{max}\frac{GLY}{Kmghmtr}}{1+\frac{SER}{Kmghmtf}+\frac{GLY}{Kmghmtr}}$

**Parameters**

The parameters used in the equations and the justifications for their starting points are shown below.

**Steady state concentrations**

Starting values of model concentrations were taken from a total of several references with values below([Buxton and Frank 1997](#_ENREF_1); [Ercan-Fang, Gannon et al. 2002](#_ENREF_2); [Konig, Bulik et al. 2012](#_ENREF_4)).

| **Steady State Species concentrations** | | | |
| --- | --- | --- | --- |
| **Species** | **Conc., mM** | **Conc range, mM** | |
| **GLC** | 2.5 | 3.5-6.9 | |
| **G6P** | 0.25 | 0.05-0.32 | |
| **F6P** | 7.73E-02 | 0.01-0.1 | |
| **F16BP** | 1.55E-01 | 0.016-0.030 | |
| **GA3P** | 2.00E-03 | 0.001-0.28 | |
| **DHAP** | 4.14E-02 | 0.01-0.1 | |
| **13BPG*** | 1.00E-01 |  | |
| **3PG** | 0.5 | 0.05-0.41 | |
| **2PG** | 3.00E-02 | 0.007-0.05 | |
| **PEP** | 0.15 | 0.012-0.27 | |
| **PYR** | 5.00E-01 | 0.02-0.27 | |
| **LAC** | 5 | 0.1-2.5 | |
| **ATP** | 3.00E+00 | 0.5-3.5 | |
| **ADP** | 1.18E-02 | 0.5-1.4 | |
| **AMP** | 4.62E-05 | 0.04 | |
| **Pi** | 4.00E+00 | 3.6-5.7 | |
| **NADH** | 1.00E-03 | 0.03-0.05 | |
| **NAD+** | 5.49E-01 | 0.45 | |
| **PCr** | 1.00E+01 | 10.0-21.0 | |
| **O2i**** | 0.04 | 0.026-0.034 (25mmHg) | |
| **SER** | 3.00E-01 |  | |
| **GLY** | 1.30E-01 |  | |
| **pSER** | 3.00E-01 |  | |
| **pH** | 7.00E+00 |  | |
| **Extracellular concentrations** |  |  |  |
| **GLCe** | 5.00E+00 | |  |
| **LACe** | 5.00E-01 | |  |
| **SERe** | 3.00E-01 | |  |
| **GLYe** | 1.30E-01 | |  |

* estimated based on Thermodynamics

** calculated based on Henry's law of solubility of O_2_ in water at 37**°**C

**Rate constants**

Starting rate constants are shown below. The K_m_ values for substrates in glycolysis are set equal to the steady state substrate concentrations in single-substrate reactions and equal to the product of substrate concentrations in multisubstrate reactions. K_m_ values are reported in units of mM. Vmax values are reported in units of mM/hr.

| **Enzyme** | **Parameter/value** | |  |  |  |  |  |  |
| --- | --- | --- | --- | --- | --- | --- | --- | --- |
| **GLUT** | ***Vmaxtr*** | ***Kmglc*** |  |  |  |  |  |  |
|  | 100 | 2.1 |  |  |  |  |  |  |
| **HK** | ***Vmaxf*** | ***Kmf*** | ***Kmr*** | ***Ki*** |  |  |  |  |
|  | 176 | 7.5 | 2.94E-10 | 0.2 |  |  |  |  |
| **PGI** | ***Vmaxf*** | ***Kmf*** | ***Kmr*** |  |  |  |  |  |
|  | 858 | 0.25 | 7.73E-02 |  |  |  |  |  |
| **PFK** | ***Vmaxf*** | ***Kmf*** | ***Kmr*** | ***Ka*** |  |  |  |  |
|  | 1769 | 0.23 | 1.82E-10 | 0.001 |  |  |  |  |
| **ALD** | ***Vmaxf*** | ***Kmf*** | ***Kmr*** |  |  |  |  |  |
|  | 321 | 0.16 | 8.29E-05 |  |  |  |  |  |
| **TPI** | ***Vmaxf*** | ***Kmf*** | ***Kmr*** |  |  |  |  |  |
|  | 859 | 0.04 | 0.002 |  |  |  |  |  |
| **GAPDH** | ***Vmaxf*** | ***Kmnad*** | ***Kmnadh*** | ***Kmgap*** | ***Kmpi*** | ***Kmbpg*** | ***Kmf*** | ***Kmr*** |
|  | 781 | 0.55 | 0.001 | 2.00E-03 | 4 | 1.00E-01 | 4.392e--3 | 1.00E-11 |
| **PGK** | ***Vmaxf*** | ***Kmf*** | ***Kmr*** |  |  |  |  |  |
|  | 221 | 1.18E-03 | 1.5 |  |  |  |  |  |
| **PGM** | ***Vmaxf*** | ***Kmf*** | ***Kmr*** |  |  |  |  |  |
|  | 527.895 | 0.5 | 0.03 |  |  |  |  |  |
| **ENO** | ***Vmaxf*** | ***Kmf*** | ***Kmr*** |  |  |  |  |  |
|  | 1340.089 | 0.03 | 0.15 |  |  |  |  |  |
| **PK** | ***Vmaxf*** | ***Kmf*** | ***Kmr*** | ***Ka*** |  |  |  |  |
|  | 211.525 | 1.77E-03 | 0.15 | 0.5 |  |  |  |  |
| **LDH** | ***Vmaxf*** | ***Kmnadh*** | ***Kmnad*** | ***Kmpyr*** | ***Kmlac*** | ***Kmf*** | ***Kmr*** |  |
|  | 434 | 0.001 | 0.549 | 0.5 | 5 | 5.00E-04 | 2.745 |  |
| **MCT** | ***Vmaxtr*** | ***Kmlac*** |  |  |  |  |  |  |
|  | 60 | 3 |  |  |  |  |  |  |
| **OxPhos** | ***Vmax*** | ***Kmpyr*** | ***Kmo2*** | ***Kmadp*** |  |  |  |  |
|  | 8.4 | 0.001 | 0.005 | 0.005 |  |  |  |  |
| **ATPase** | ***Vmaxf*** | ***Kmf*** | ***Kmr*** |  |  |  |  |  |
|  | 390 | 3 | 4.71E-09 |  |  |  |  |  |
| **CKase** | ***Vmaxf*** | ***Kmf*** | ***Kmr*** |  |  |  |  |  |
|  | 1000 | 5 | 2 |  |  |  |  |  |
| **AKase** | ***Vmaxf*** | ***Kmf*** | ***Kmr*** |  |  |  |  |  |
|  | 2000 | 5 | 2 |  |  |  |  |  |
| **O2transp** | ***ko2*** |  |  |  |  |  |  |  |
|  | 164 |  |  |  |  |  |  |  |
| **PHGDH** | ***Vmaxf*** | ***Km3pg*** | ***Kmnad*** | ***Kmser*** | ***Kmnadh*** | ***Kmf*** | ***Kmr*** |  |
|  | 0 | 0.5 | 0.5 | 0.1 | 0.01 | 0.25 | 1.00E-10 |  |
| **GHMT** | ***Vmaxf*** | ***Kmf*** | ***Kmr*** |  |  |  |  |  |
|  | 0 | 1.5 | 0.5 |  |  |  |  |  |
| ***Other parameters*** | ***nop*** | ***nfad*** | ***nnad*** | ***nox*** |  |  |  |  |
|  | 12.5 | 1.5 | 2.5 | 3 |  |  |  |  |

**Cell uptake and release fluxes**

Typical uptake and release fluxes were considered([Shestov, Mancuso et al. 2013](#_ENREF_6)).

| Cell Uptake-Release Fluxes mM/h |  |
| --- | --- |
| Fglc | 80 |
| Fmpc | 8.8 |
| Ftca | 10.7 |
| Fldh | 150 |
| Fltr | 150 |
| CMRO2 | 34 |
| Fatp(cyt) | 150 |
| Fatp(mit) | 174 |
| WE | 14.6 |

**Thermodynamics**

Standard Gibbs Free Energies (dG) were used to constrain fluxes based on Haldane relationships([Goldberg, Tewari et al. 2004](#_ENREF_3); [Li, Wu et al. 2011](#_ENREF_5)).

| Reaction | dG, kJ/mol |
| --- | --- |
| HK | -19.22 |
| PGI | 2.78 |
| PFK | -15.62 |
| ALD | 24.64 |
| TPI | 7.57 |
| GAPDH | 2.6 |
| PGK | -21.6 |
| PGM | 6.35 |
| ENO | -4.47 |
| PK | -27.18 |
| LDH | -23.9 |
| CK | -12.5 |
| AK | 0 |
| ATPase | -32.42 |

**Supplementary References to Materials and Methods**

Buxton, R. B. and L. R. Frank (1997). "A model for the coupling between cerebral blood flow and oxygen metabolism during neural stimulation." J Cereb Blood Flow Metab **17**(1): 64-72.

Ercan-Fang, N., M. C. Gannon, et al. (2002). "Integrated effects of multiple modulators on human liver glycogen phosphorylase a." Am J Physiol Endocrinol Metab **283**(1): E29-37.

Goldberg, R. N., Y. B. Tewari, et al. (2004). "Thermodynamics of enzyme-catalyzed reactions--a database for quantitative biochemistry." Bioinformatics **20**(16): 2874-2877.

Konig, M., S. Bulik, et al. (2012). "Quantifying the contribution of the liver to glucose homeostasis: a detailed kinetic model of human hepatic glucose metabolism." PLoS Comput Biol **8**(6): e1002577.

Li, X., F. Wu, et al. (2011). "A database of thermodynamic properties of the reactions of glycolysis, the tricarboxylic acid cycle, and the pentose phosphate pathway." Database (Oxford) **2011**: bar005.

Shestov, A. A., A. Mancuso, et al. (2013). "Metabolic network analysis of DB1 melanoma cells: how much energy is derived from aerobic glycolysis?" Adv Exp Med Biol **765**: 265-271.
